# Efficacy of electroacupuncture compared with transcutaneous electric nerve stimulation for functional constipation: Study protocol for a randomized, controlled trial

**DOI:** 10.1097/MD.0000000000010692

**Published:** 2018-05-11

**Authors:** Yuxiao Zeng, Xuecheng Zhang, Jing Zhou, Xinwei Wang, Ruimin Jiao, Zhishun Liu

**Affiliations:** a Department of Acupuncture, Guang’anmen Hospital, China Academy of Chinese Medical Sciences; b China Academy of Chinese Medical Sciences; c Beijing University of Chinese Medicine, Beijing, China.

**Keywords:** acupuncture, functional constipation, randomized controlled trial, transcutaneous electric nerve stimulation

## Abstract

**Background:**

To treat functional constipation, both electroacupuncture (EA) therapy and transcutaneous electric nerve stimulation (TENS) are safe and effective. However, no head-to-head comparison trial has been conducted. This trial compares the efficacy of electroacupuncture relative to transcutaneous electric nerve stimulation for functional constipation.

**Methods:**

Individuals with functional constipation will be randomly allocated to receive either EA or TENS (n = 51, each), 3 times per week for 8 weeks. The primary outcome is the percentage of participants with an average increase from baseline of 1 or more complete spontaneous bowel movements at week 8. The secondary outcome measures are the following: at the time of visits, changes in the number of complete spontaneous bowel movements, number of spontaneous bowel movements, stool character, difficulty in defecation, patients’ assessment of quality of life regarding constipation (self-report questionnaire), and use of auxiliary defecation methods.

**Discussion:**

The results of this trial should verify whether EA is more efficacious than TENS for relieving symptoms of functional constipation. The major limitation of the study is the lack of blinding of the participants and acupuncturist.

## 1 Introduction

Functional constipation is a common chronic gastroenteric disease. According to epidemiological data, the prevalence of constipation in the worldwide population is 0.7% to 79.0%, and 19.2%, 19.7%, and 10.8% in Europe, Oceania, and Asia, respectively.^[1]^ There are currently 3 broad categories of therapies for functional constipation: nonpharmaceutical, pharmaceutical, and surgical. The most widely applied therapy is nonpharmaceutical, via lifestyle adjustment to establish good habits of defecation. Pharmaceutical treatments are used to relieve symptoms, but constipation tends to recur,^[2,3]^ and the side effects of most medications cannot be ignored.^[4–6]^ Surgical treatment has strict indications and is performed only in exceptional cases ^[7]^ or reserved for extreme cases of colonic inertia;^[8]^ surgery to treat constipation is not routine.

In recent years, acupuncture to correct constipation has attracted attention. A systematic review showed that acupuncture is effective for this purpose.^[9]^ In addition, a multicenter randomized controlled trial involving 1075 subjects showed that electroacupuncture (EA) was safe and effective for chronic severe functional constipation.^[10]^

To treat constipation, transcutaneous electrical nerve stimulation (TENS) delivers a targeted electrical current to specific areas or the spinal ganglion segment of the body surface that regulates peristalsis of the colon.^[11]^ According to a systematic review, TENS can accelerate colonic transit and has a certain therapeutic effect on functional constipation.^[12]^

Theoretically, the therapeutic effect of EA for treating constipation may be better than that of TENS, since the latter stimulates only the skin, while the EA needle delivers current at a deeper level. TENS may be less able than EA to overcome the electrical resistance of the skin, subcutaneous tissues, and muscles.

In this study, we will compare the efficacy and safety of EA relative to TENS (control) for the treatment of functional constipation.

## 2 Methods

### 2.1 Study design

**Figure 1:**
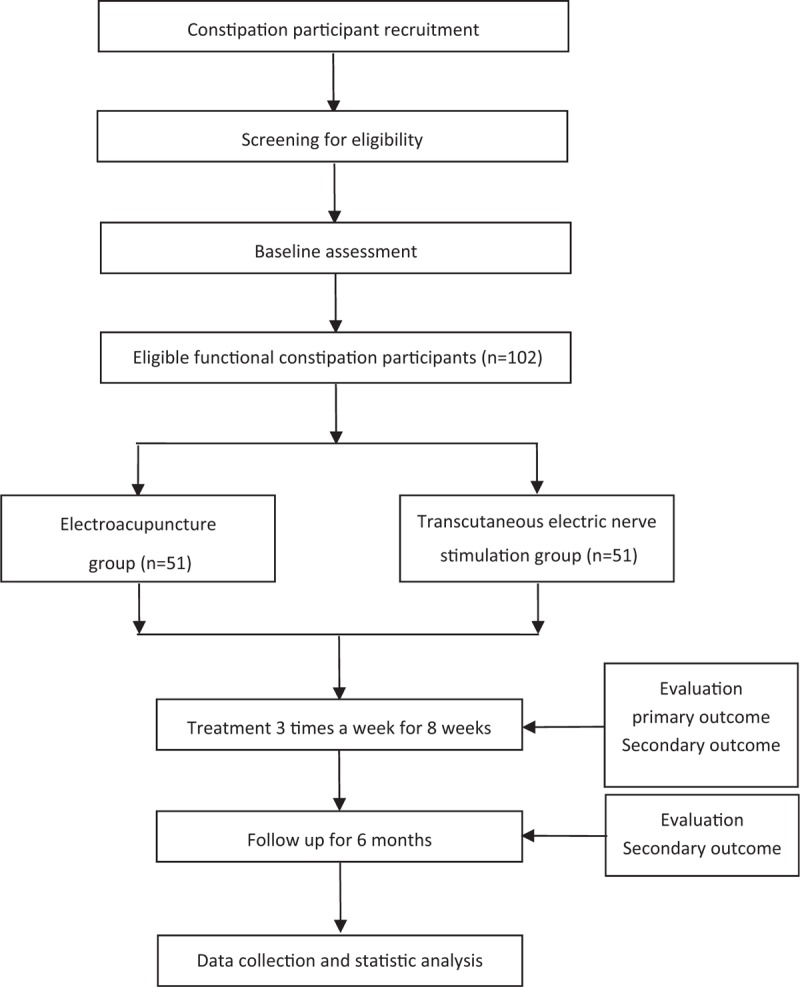
Study design schedule-time frame of the trial.

**Figure 2:**
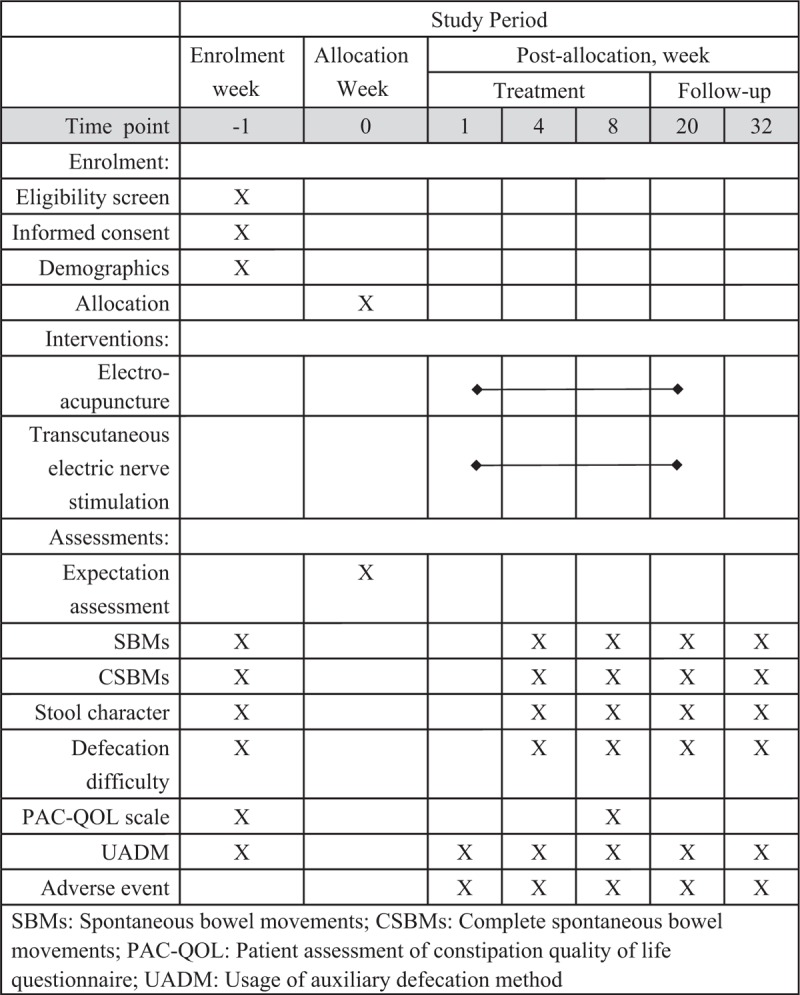
Standard protocol items: recommendations for interventional trials (SPIRIT).

This is a randomized parallel-group controlled trial. The flowchart and time point of the trial assessment are presented in Figs. 1 and 2, respectively. The trial protocol conforms to the principles of the Declaration of Helsinki and has been approved by the Ethics Committee of Guang’anmen Hospital of China Academy of Chinese Medical Sciences (2017-059-KY-01). All modifications of this protocol will be submitted to the Ethics Committee of Guang’anmen Hospital. And this trial has been registered at clinical trials.gov (NCT03391635). All eligible participants will be required to provide a sign informed consent before being randomly allocated.

### 2.2 Study setting and recruitment

This study will be conducted from July 2018 to December 2020 in Guang’anmen Hospital, China Academy of Chinese Medical Sciences. A total of 102 participants with functional constipation will be recruited openly through posters, hospital websites, and networks. A postgraduate is responsible for consultation with the participants and screening, filling out the screening form, recording demographic data, history of diagnosis and treatment, inclusion and exclusion criteria, and other basic data of the participants. The qualified participants will undergo a baseline evaluation for 1 week.

For inclusion in this study, participants will conform to the following criteria: with functional constipation as defined by the Rome III criteria;^[13]^ specific symptoms including laborious defecation or <3 times per week, hard stool, a sense of obstruction, needing an auxiliary defecation method; aged 18 to 75 years; volunteered to participate and provided signed informed consent.

Potential participants with any of the following will be excluded from this study: irritable bowel syndrome; organic constipation or secondary constipation caused by endocrine, metabolic, neurogenic, or postoperative diseases; combined with severe heart, liver, or kidney damage; cognitive impairment, aphasia, or mental disorders; inability to cooperate with the examination or treatment; pregnant or lactating; abdominal aortic aneurysm; abnormal enlargement of the liver or spleen; blood coagulation dysfunction or chronic use of anticoagulants such as Warfarin and Heparin; or with an installed cardiac pacemaker.

### 2.3 Randomization

The randomization process will be performed using Statistics Analysis System (SAS) software in the office of the clinical drug test agency of Guang’anmen Hospital. The random numbers and groups will be sealed in opaque envelopes for random allocation and concealment. The acupuncturists will obtain the information of the random numbers and group assignment by opening these envelopes.

### 2.4 Blinding

The evaluator, data processor, statisticians, and other researchers who are not involved in the treatment process will be blinded to the treatment.

### 2.5 Interventions

#### 2.5.1 Electroacupuncture (EA) group

The acupoints are bilateral Tianshu (ST [stomach] 25), Fujie (SP [spleen] 14), and Shangjuxu (ST37), according to the classic acupuncture point prescriptions within the Traditional Chinese Medicine system (National Standard of People's Republic of China [GB/T 12346-2006] Nomenclature and Location of Acupuncture Points in 2006).^[14]^

During the EA, the participant is supine. After routinely sterilizing the skin, stainless filiform needles (0.30 × 50 mm or 0.30 × 75 mm, Huatuo Brand, Suzhou Medical Appliance, China) will be inserted into bilateral ST25 and SP14 vertically and slowly until touching the abdominal muscle layer. The bilateral ST37 will be inserted to a depth of 25 to 30 mm with the manipulation of steady small lifting, thrusting, and twirling 3 times, until the subject experiences a sensation of de qi (sourness, numbness, and heaviness). An electric acupuncture apparatus (SDZ-V EA; Huatuo, Suzhou, China) will be applied to bilateral ST25, SP14, and ST37 with a dilatational wave of 2/10 Hz and an electric current intensity of 0.1 to 1.0 mA, depending on the participant's tolerance, with a slight tremor of the participant's muscles.

Each session will last for 30 minutes. Participants will be treated 3 ×/wk for 8 weeks. Each participant will undergo 24 sessions.

#### 2.5.2 Transcutaneous electric nerve stimulation (TENS) group

The acupoints, location, and electroacupuncture apparatus of the TENS control group will be the same as for the EA group, the only difference being that the front end of the wire of the electric acupuncture apparatus is changed to a skin electrode patch without acupuncture. The participant will be supine, and the skin routinely sterilized. Skin electrode patches (50 × 50 mm) will be placed on bilateral ST25, SP14, and ST37 with a dilatational wave of 2/10 Hz and an electric current intensity of 2 to 5 mA,^[15,16]^ depending on the participant's tolerance.

The frequency and treatment sessions will be the same as for the EA group.

### 2.6 Outcome measurement

#### 2.6.1 Primary outcome

The primary outcome is the percentage of participants with an average increase of 1 or more complete spontaneous bowel movements (CSBMs) during the interval from baseline through week 8.

#### 2.6.2 Secondary outcomes

The secondary outcome measures are the following 7 items: percentage of participants with an average increase from baseline of at least 1 CSBM at weeks 4, 20, and 32; change in the number of CSBMs at weeks 4, 8, 20, and 32; change in the number of spontaneous bowel movements (SBMs) at weeks 4, 8, 20, and 32; percentage of participants with type 3 or type 4 stool at weeks 4, 8, 20, and 32, as defined by the Bristol scale;^[17]^ change in average score for difficult defecation at weeks 4, 8, 20, and 32; change in total score on the PAC-QOL questionnaire ^[18]^ at the end of week 8, relative to baseline; and the percentage of participants using cathartics, and the average amount of cathartics, at weeks −1, 4, 8, 20, and 32.

The participants will be asked to record a diary of defecation every day during weeks −1, 4, 8, 20, and 32 after randomization. The recordings are to include bowel movements, SBMs, CSBMs, the stool character, degree of difficulty in defecation, and the use of auxiliary defecation methods. All adverse events that occur during the trial will be recorded. Serious adverse events should be reported to the principal investigator immediately.

The participants’ expectations will be assessed before the intervention with 4 brief questions for which the answers chosen may be “Yes,” “No,” or “Unclear,” as follows: “Do you think EA is effective for treating illness?”; “Do you think EA will help improve your symptoms?”; “Do you think TENS is effective for treating illness?”; “Do you think TENS will help improve your symptoms?”. In addition, a fifth question (“Which kind of treatment do you prefer?”) may be answered as “EA,” “TENS,” or “Does not matter.”

### 2.7 Statistics

#### 2.7.1 Sample size

The sample size was calculated based on the primary outcome. According to a previous related clinical study, the percentage of patients in the EA group, whose CSBMs increased by at least once per week from the baseline, was 69.7% at week 8, while that of the sham EA group was 44.6%.^[19]^ We assume that the corresponding indicator in the present study may be 70% in the EA group and 40% in the TENS group, based on relevant literature reports at home and abroad.^[19–21]^

According to the formula for estimating sample size to evaluate a difference between 2 groups (with 80% power to detect a difference of 30%) in a 2-sided test, each group size should be 42, with an alpha risk of 5% and a beta risk of 20%. Assuming a 20% expulsion rate, a total of 102 participants (51 participants in each group) will be required for the study.

#### 2.7.2 Statistical analysis

The statistical analyses will be performed using the SPSS 20.0 statistical software. Continuous data will be represented by the mean, standard deviation, median, minimum value, and maximum value. Categorical data will be represented by percentage. For the data with normal distribution, the *t* test or rank-sum test will be used for continuous data, and a nonparametric test or *χ*^2^ test for categorical data. A rank-sum test or *χ*^2^ test will be used to analyze the difference between 2 independent samples. Statistical analyses will be 2-sided tests. A *P* value <.05 will be considered statistically significant.

#### 2.7.3 Data collection, management, and monitoring

The original data collected during this trial must be traceable, such as with a case report form and informed consent form. The researchers will manually fill in the case report form, collect data regularly, and use the double-input method to enter data. All data of these participants will be preserved safely and the personal data will only be used for this study. The data management and the whole process of this study will be closely supervised.

## 3 Discussion

The purpose of this study is to compare the therapeutic effect of EA relative to TENS for the treatment of functional constipation. Both EA and TENS are nonpharmaceutical, and considered beneficial as low-frequency electrical stimulation therapies. In theory, EA may have a better therapeutic effect than TENS. Although the acupoints, electric impulse frequency, and waveforms of the 2 treatments are the same, the output current intensity of TENS is several times that of EA. Participants in the TENS group cannot feel a current that is less than 1 mA, because of the higher electrical resistance of the skin, subcutaneous tissues, and muscles. However, a current intensity of 1 mA through the EA needle is obviously felt, and may be unbearable beyond that intensity. The current intensities of the 2 groups are different, because the intensity of the electric current depends on the maximum strength that the participant can tolerate.

One of the limitations of this trial design is that the patients and acupuncturists will not be blinded to the treatment. However, the curative effect evaluator, data processor, and statisticians will be blinded to the treatment allocation, to minimize the potential bias.

The results of this trial should verify whether EA is more efficacious than TENS for relieving symptoms of functional constipation.

## Acknowledgments

The authors of this manuscript would like to give their sincere thanks to the editors of Medjaden Bioscience Limited for help editing the manuscript.

## Author contributions

XZ and ZL conceived the concept and design of the study. XZ drafted and edited the final paper for submission. ZL reviewed and amended the final paper. XZ, JZ, XW, and RJ prepared the related information sheets, scales, and consent forms. All authors approved the final manuscript.

**Investigation:** Yuxiao Zeng, Xuecheng Zhang, Jing Zhou, Xinwei Wang, Ruimin Jiao.

**Methodology:** Yuxiao Zeng.

**Writing – original draft:** Yuxiao Zeng.

**Writing – review & editing:** Yuxiao Zeng.

**Conceptualization:** Zhishun Liu.

**Funding acquisition:** Zhishun Liu.

**Supervision:** Zhishun Liu.

**Project administration:** Xuecheng Zhang, Jing Zhou, Xinwei Wang, Ruimin Jiao.

**Resources:** Xuecheng Zhang, Jing Zhou, Xinwei Wang, Ruimin Jiao.
